# Clonal copy-number mosaicism in autoreactive T lymphocytes in diabetic NOD mice

**DOI:** 10.1101/gr.247882.118

**Published:** 2019-12

**Authors:** Maha Alriyami, Luc Marchand, Quan Li, Xiaoyu Du, Martin Olivier, Constantin Polychronakos

**Affiliations:** 1 The Endocrine Genetics Laboratory, Child Health and Human Development Program and Department of Pediatrics, McGill University Health Centre Research Institute, Montreal, Quebec H3H 1P3, Canada;; 2 Department of Biochemistry, College of Medicine and Health Sciences, Sultan Qaboos University, 123, Muscat, Oman;; 3 Princess Margaret Cancer Centre, University Health Network, Toronto, Ontario, ON M5G 2C1, Canada;; 4 Departments of Medicine, Microbiology, and Immunology, McGill University Health Centre Research Institute, Montreal, Quebec H3H 1P3, Canada

## Abstract

Concordance for type 1 diabetes (T1D) is far from 100% in monozygotic twins and in inbred nonobese diabetic (NOD) mice, despite genetic identity and shared environment during incidence peak years. This points to stochastic determinants, such as postzygotic mutations (PZMs) in the expanding antigen-specific autoreactive T cell lineages, by analogy to their role in the expanding tumor lineage in cancer. Using comparative genomic hybridization of DNA from pancreatic lymph-node memory CD4^+^ T cells of 25 diabetic NOD mice, we found lymphocyte-exclusive mosaic somatic copy-number aberrations (CNAs) with highly nonrandom independent involvement of the same gene(s) across different mice, some with an autoimmunity association (e.g., *Ilf3* and *Dgka*). We confirmed genes of interest using the gold standard approach for CNA quantification, multiplex ligation-dependent probe amplification (MLPA), as an independent method. As controls, we examined lymphocytes expanded during normal host defense (17 NOD and BALB/c mice infected with *Leishmania major* parasite). Here, CNAs found were fewer and significantly smaller compared to those in autoreactive cells (*P* = 0.0019). We determined a low T cell clonality for our samples suggesting a prethymic formation of these CNAs. In this study, we describe a novel, unexplored phenomenon of a potential causal contribution of PZMs in autoreactive T cells in T1D pathogenesis. We expect that exploration of point mutations and studies in human T cells will enable the further delineation of driver genes to target for functional studies. Our findings challenge the classical notions of autoimmunity and open conceptual avenues toward individualized prevention and therapeutics.

Type 1 diabetes (T1D) is an autoimmune disease caused by targeted destruction of the insulin-producing beta cells through infiltration of autoreactive T lymphocytes (Polychronakos and Li 2011). The disease is antigen-specific, in which this autoimmune process of infiltration destroys only the insulin-producing beta cells. Although T1D is known to depend on both inherited susceptibility and environmental factors, these alone may not explain all of the disease. Concordance in monozygotic twins is only 65% and age of onset can differ by several decades (Redondo et al. 2008). A shared environment in early life, at the onset in the first twin, also raises some doubt about whether environment accounts for this difference (Knip et al. 2005). Similarly, in the inbred nonobese diabetic (NOD) mouse model, not all females develop the disease and males have an incidence of <50% despite being genetically identical and kept in a standardized environment (Makino et al. 1980). These observations suggest stochastic events. One plausible such event could consist of postzygotic genetic changes in the expanding antigen-specific autoreactive T cell lineages.

The hypothesis suggesting the contribution of postzygotic mutations (PZM) in the pathogenesis of autoimmune diseases was first put forward in 1972 by Burnet (Burnet 1972), who proposed that the stochastic nature of autoimmune diseases might be caused by a combination of germline and somatic mutations that interrupt normal mechanisms for eliminating self-reactive lymphocytes and causing the development of “forbidden clones.” The hypothesis was proposed again in 2007 by Goodnow (Goodnow 2007), who hypothesized a major contribution of PZMs in the pathogenesis of autoimmune diseases, in a paradigm similar to the pathogenesis of cancer. In 2004, Holzelova (Holzelova et al. 2004) detected heterozygous dominant *Fas* mutations in a fraction of T cells of sporadic cases of the autoimmune lymphoproliferative syndrome (ALPS) without the development of lymphoma. This condition follows the conventional two-hit cancer model, with the somatic mutation compounding one inherited on the opposite allele (Dowdell et al. 2010; Magerus-Chatinet et al. 2011). Here, we hypothesized that the phenomenon applies more generally in autoimmunity and involves modulation (not necessarily complete loss of function) of multiple genes.

In blood cells, PZMs (copy-number or point mutations) result in a mosaic state that can occasionally be detected in the peripheral whole blood of healthy individuals (Forsberg et al. 2012; Jacobs et al. 2012; Laurie et al. 2012). These findings almost certainly underestimate the frequency of these events in the general population, as peripheral whole-blood is a heterogeneous mixture, within which the PZM mosaicism is too low to cause a clinical phenotype or to be detectable by conventional methods (Jacobs et al. 2012). PZM frequency increases with age, indicating that their rise to detectable levels is due to some proliferation/survival advantage. The contribution of copy-number somatic mutations in the pathogenesis of cancer has been established and has enabled therapeutic advances.

In this study, we investigated the PZM hypothesis as part of the cause of diabetes in NOD mice, a model of spontaneous insulitis that closely recapitulates the destruction of the beta cells by autoreactive CD4^+^ and CD8^+^ T cells in T1D (Polychronakos and Li 2011; Pearson et al. 2016). Similar to human T1D, diabetes in NOD mice is caused by a combination of polygenic inheritance and environmental factors (Polychronakos and Li 2011; Pearson et al. 2016). Female mice are predominantly affected (90%–100%), while males develop it at an older age with lower frequency. We hypothesize that PZMs cause T cells to escape self-tolerance checkpoints, with expansion of autoreactive lineages. As a first step, we focused on copy-number mutations, since they are known to occur in blood cells and have more obvious functional effects.

## Results

To test the hypothesis, we compared the genomes of the autoreactive lymphocytes (memory cells from pancreatic lymph nodes [PLN]) to the germline genome (tail DNA) of the diabetic mice by comparative genomic hybridization (CGH) to identify somatic copy-number mutations in the proliferating immune cells.

**Figure 1.**
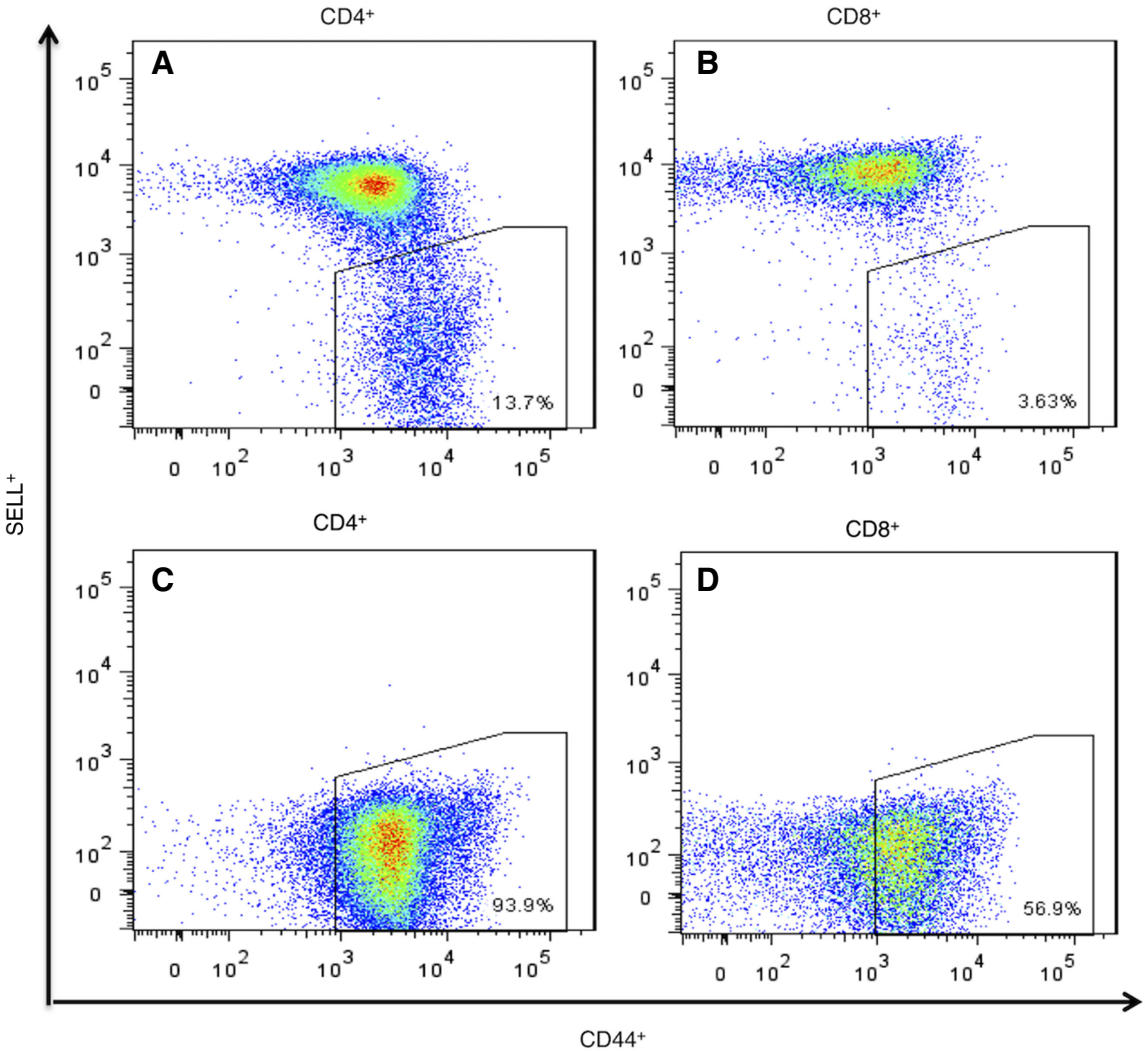
Flow-sorting of memory CD4^+^ and CD8^+^ T cells from NOD mouse PLNs using the established surface-marker phenotype SELL^neg^/CD44^hi^. (*A*,*B*) Normal profile observed in most samples of CD4^+^ and CD8^+^ T cells (*n* = 19). (*C*,*D*) Profile of sorted memory cells in six animals (*n* = 6) in which all cells were SELL^neg^ with CD44^+^ in the lower part of the range for memory cells.

In our vivarium, 90%–100% of the female NOD mice and about 50% of males become diabetic (Supplemental Fig. S1). We euthanized NOD mice, within 2–4 d of hyperglycemia onset and obtained the PLNs and a tail-clip sample. Memory CD4^+^ cells from PLNs were isolated on a FACSAria sorter based on the SELL^neg^/CD44^hi^ phenotype (Fig. 1A,B). Average yield was 124,672 CD4^+^ cells per mouse. In six out of the 25 mice examined, all T cells were negative for selectin, lymphocyte (SELL, also known as CD62L), indicating a strong preponderance of memory cells in the PLN (Fig. 1C,D). DNA from these cells was analyzed by CGH, with tail DNA as reference.

### Positive and negative controls

**Figure 2.**
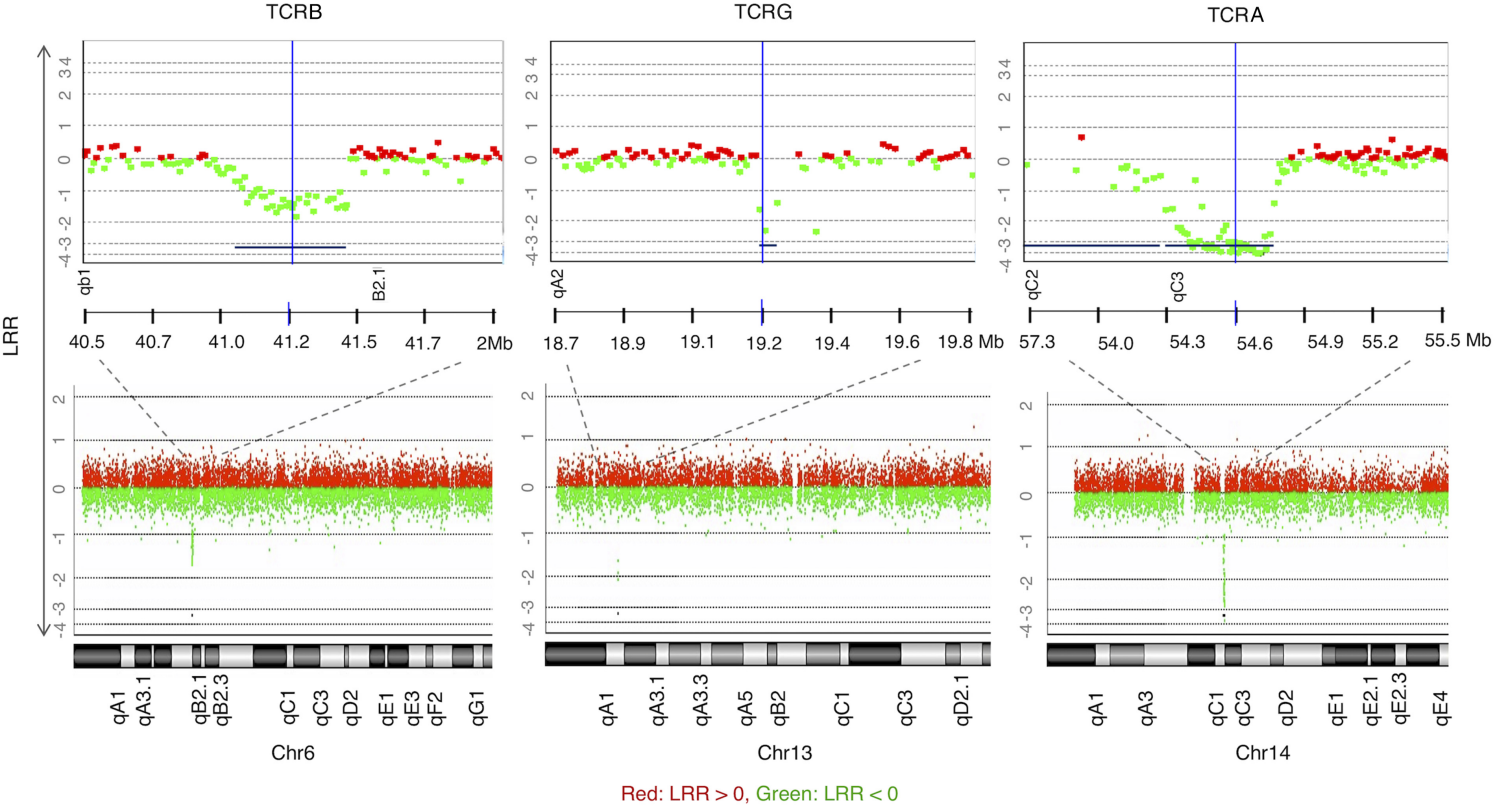
CGH array results demonstrating positive controls for T cell CNAs observed in all samples (to illustrate, M1 is shown here), copy loss (regions represented by lines *below* the graph) at regions of TCR genomic rearrangements of the β-chain on Chr 6, γ-chain on Chr 13, and α-chain on Chr 14 (DLRSpread value: M1: 0.21). LRR (log relative ratio) is the base 2 log of the Cy3/Cy5 ratio.

CGH data of all samples revealed the expected deletions at the T cell receptor loci (B, G, and A–D subunits) due to genomic rearrangements to create TCR diversity in lymphocytes—a naturally occurring positive control (Fig. 2).

Technical controls evaluating the specificity of the method demonstrated an absence of amplification artifacts in CNA calls. As expected, no CNAs were called when the same sample was used as test and reference due to the identicality of the two samples (Sample M26; DLRSpead value: 0.17). Moreover, no CNAs were called for the duplicate samples M9 and M9B (DLRSpead value: 0.18 and 0.21, respectively).

**Figure 3.**
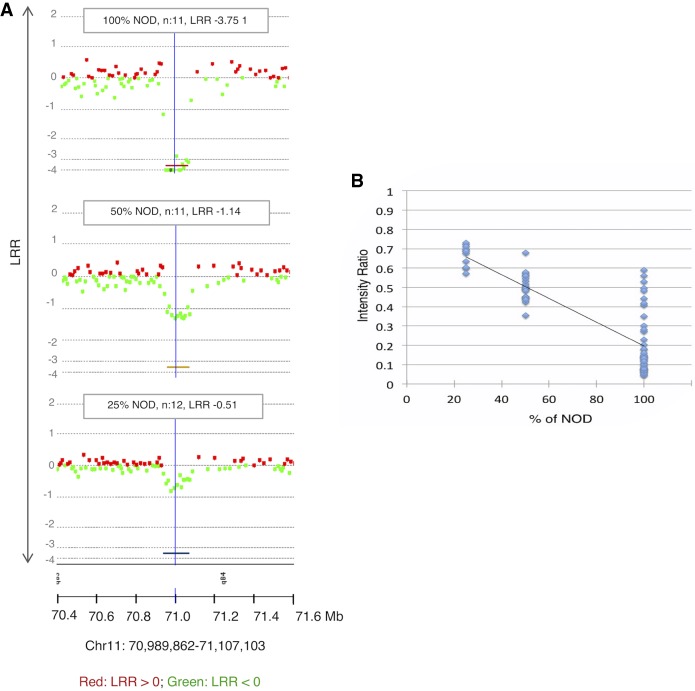
Detection of mosaicism. (*A*) Example of results of the three mixtures of germline NOD and C57BL6/J at the indicated percentages against 100% C57BL6/J as a reference. A known germline CNV between the two strains at Chr 11: 70,989,862–71,107,103 (region represented by colored lines *below* the charts) is significant even at 25%. (*B*) The intensity ratio of NOD copy losses is significantly lower than the baseline represented by 1, allowing estimates of mosaicism even <25% (DLRSpread values: 100% NOD: 0.23; 50% NOD: 0.20; 25% NOD: 0.15); *n* = number of probes.

To evaluate the sensitivity of CGH for mosaic CNAs, we evaluated mixtures of NOD and C57BL6/J tail DNA against C57BL6/J as a reference. Known CNVs between the two strains were detectable and statistically significant even at 25% mosaicism (Fig. 3A) and correlated linearly with the Cy3/Cy5 ratio (Fig. 3B; Supplemental Data S1), allowing a rough estimation of mosaicism.

### Recurrent postzygotic copy number changes in pancreatic lymph nodes of T cells of different mice

The 25 tested mice (22 females and three males) (Supplemental Table S1) had a total of 403 somatic CNAs (Supplemental Data S2, S3). Four of the mice were also investigated for CNAs in CD4^+^ from the peripheral LN (M16L, M17L, M20L, and M21L). No CNAs were called in samples obtained from the peripheral LN of these mice. The number of CNAs per mouse varied widely. M23 and M25 (two of the mice with memory-only cells in PLNs) had an unusually large number of CNAs.

**Figure 4.**
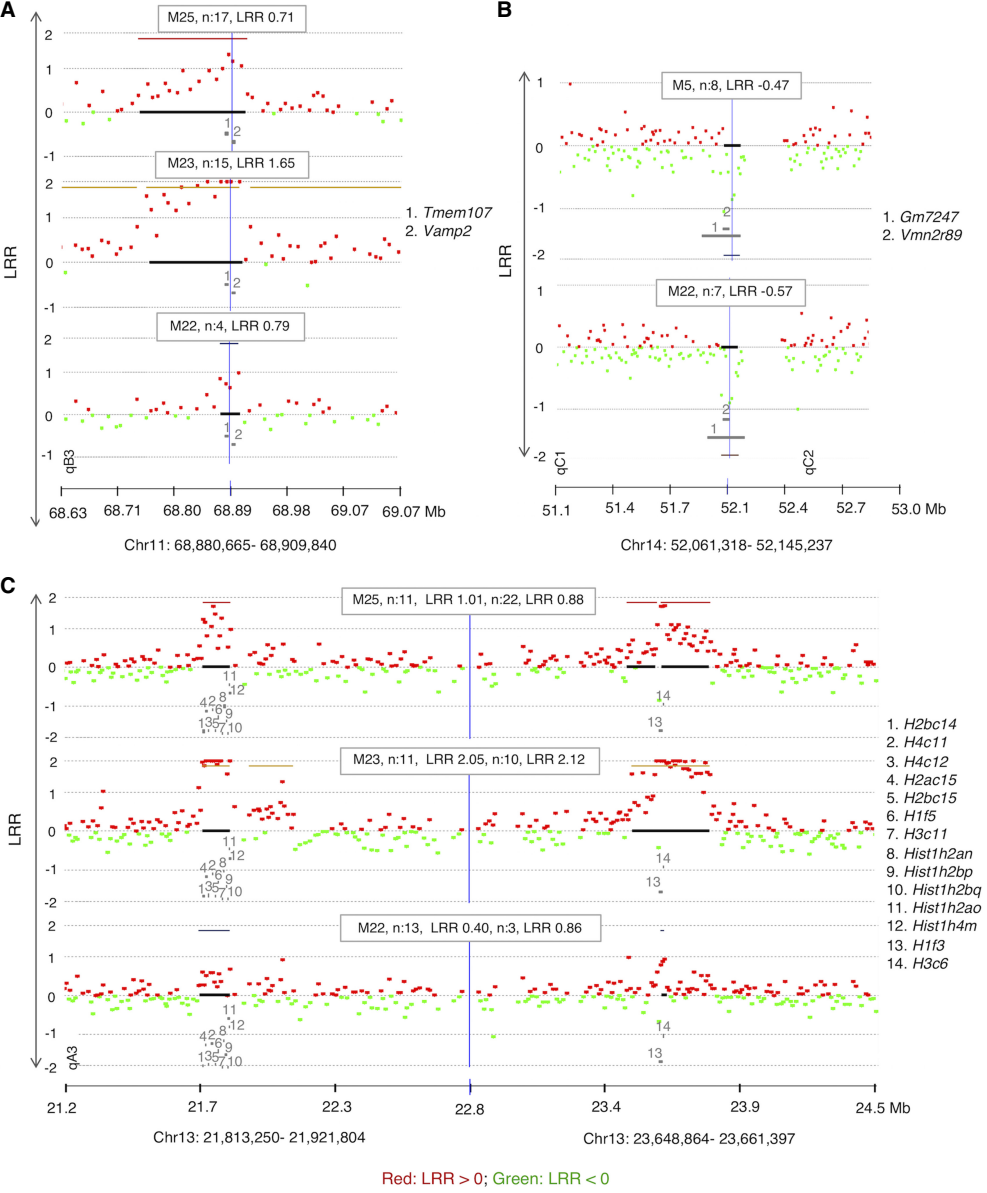
CGH array results showing recurrent CNAs in memory cells from PLNs of diabetic mice (n: number of affected probes, location of genes is indicated by numbers). (*A*) A recurrent copy gain in cells of three mice spanning several genes including *Tmem107.* (*B*) Recurrent copy loss spanning two undocumented genes (*Gm7247* and *Vmn2r89*). (*C)* An independent recurrence of copy gain in three different mice that are spanning two Histone families’ loci. The formula to calculate the percentage of mosaicism of deletions is 100 **×**(1–2^LRR^) × 2 (example: the percentage of mosaicism for −0.25 is 31.8% mosaicism and for −0.5 is 58.6%). DLRSpread values of samples are in Table 1 and Supplemental Fig. S2.

We found a total of 83 overlaps between mice, most of them involving the two outliers, M23 and M25. Not counting CNAs exclusively between M23 and M25, we identified eight recurrent CNAs that occurred independently in two or more mice (Table 1; Fig. 4; Supplemental Fig. S3). This is very unlikely to happen by chance alone (none of these loci overlap with the CNVs that we observed between NOD and C57BL6/J). After 100,000 iterations of randomizing the genomic coordinates of the probes defining these CNAs, not a single one gave the number of recurrent CNAs observed or more (*P* < 10^−6^). The same result was found after removing M23 and M25, responsible for many of the overlaps. This nonrandom recurrence supports causal-functional importance and, in the cancer paradigm, it is considered a strong indicator of pathogenicity. Many of these overlaps involve genes related to immune function.

**Table 1.**
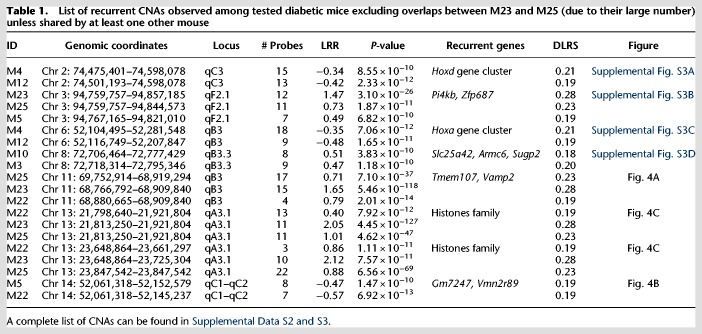
List of recurrent CNAs observed among tested diabetic mice excluding overlaps between M23 and M25 (due to their large number) unless shared by at least one other mouse

Two of the recurrent CNAs span Homeobox clusters (*Hoxd* and *Hoxa*). Both are copy losses at Chr 2: 74,474,401–74,598,078, spanning the *Hoxd* gene cluster and at Chr 6: 52,116,749–52,207,847, spanning the *Hoxa* gene cluster, both found independently in CD4^+^ T cells of two separate mice M4 and M12 (Table 1; Supplemental Fig. S3A,C). *Hox* genes control proliferation and differentiation of hematopoietic cells and have a crucial role in T cell development, being the most active cluster during early hematopoiesis (Lawrence et al. 1996; Magli et al. 1997; Taghon et al. 2002, 2003). They are implicated in leukemogenesis (Kawagoe et al. 1999) and act as tumor-suppressors (Shah and Sukumar 2010).

Another recurrent CNA is a copy gain at Chr 3: 94,767,165–94,821,010 (Table 1; Supplemental Fig. S3B) seen independently in three mice M23, M25, and M22. This CNA spans *Pi4kb* encoding phosphatidylinositol 4-kinase beta, highly expressed in hematopoietic cells (Wu et al. 2016). It phosphorylates phosphatidylinositol (PI) as the first step in the production of inositol-1, 4,5, -trisphosphate (Ins(1,4,5)P_3_), an essential signaling molecule (Heilmeyer et al. 2003). Overexpression of *Pi4kb* due to the amplification observed might be contributing to autoimmunity.

Another recurrent CNA at Chr 8: 72,706,464–72,777,429 is a copy gain in two diabetic mice (M3 and M10) (Table 1; Supplemental Fig. S3D). A common affected gene is *Armc6,* with coding sequence within the confidence interval of the breakpoint locations, indicating a high likelihood of disruption of the gene by the duplications. *Armc6* is highly conserved with Gene Ontology (GO) that suggests involvement in hematopoietic progenitor cell differentiation (GO:0002244) and in pancreatic cancer (Gress et al. 1996).

Copy gain at Chr 11: 68,880,665–68,909,840 (Table 1; Fig. 4A) was found in three mice (M22, M23, and M25). This CNA spans two genes, *Tmem107* (transmembrane protein 107) and *Vamp2* (vesicle-associated membrane protein 2)*. Tmem107* encodes a transmembrane protein 107, an uncharacterized gene that is highly expressed in T cells (http://ist.medisapiens.com). Another transmembrane encoding gene (*Igflr1*) was also included in a recurrent multicopy amplification at Chr 7: 31,316,157–31,433,964 in M25 (Supplemental Data S3) spanning several genes, including some with high expression in hematopoietic cells, such as *Igflr1, Lin37, Cox6b1*, and *Rbm42* (Wu et al. 2016). *Igflr1* encodes the transmembrane protein IGF-like family receptor 1 that is exclusively expressed in hematopoietic cells (Wu et al. 2016) and, in mice, primarily on T cells (Lobito et al. 2011). This gene is also uncharacterized, but it is structurally similar to the tumor necrosis factor receptor family (Lobito et al. 2011), and a potential inflammatory role was suggested for its encoded protein (Lobito et al. 2011).

A recurrent CNA at Chr 14: 52,061,318–52,145,237 in mice M5 and M22 is a copy loss that spans two unstudied genes *Gm7247* and *Vmn2r89* (Table 1; Fig. 4B). These two genes are also spanned by copy losses in mice M6 and M25 that were called by ADM-2 but narrowly missed the CBS cut-off (Supplemental Fig. S4A).

Two CNAs in three of the mice with the unstable genome (M22, M23, and M25) are copy gains at two histone loci at Chr 13: 21,813,250–21,921,804 and Chr 13: 23,648,864–23,661,397 (Table 1; Fig. 4C), spanning two clusters of highly homologous genes that encode nuclear proteins collectively referred to as Histone H1, H2, and H3. The histone loci are separated by a normal copy-number stretch encoding other genes. In human CD4^+^ T cells, TCR activation induces a distinctive promoter histone profile that directs the transcriptional activation of *Fasl* leading to apoptosis (Ghare et al. 2014).

Finally, the last recurrent CNA is a copy loss at Chr 5: 23,846,753–24,135,248 found independently in M16 and M12 (Supplemental Fig. S4B). It is mentioned because it includes an important candidate gene, despite the fact that it missed (*P* = 0.0018) the strict significance threshold in one of the two algorithms (CBS) in one of the two mice (M16). This CNA spans several genes that are highly expressed in hematopoietic cells, including *Kcnh2*, *Fastk*, *Tmub1,* and *Chpf2* (Wu et al. 2016). *Fastk* encodes a protein that belongs to the serine/threonine protein kinase family, and it is a potent activator of lymphocytes apoptosis (Tian et al. 1995). Moreover, it has been shown that this protein is highly activated during FAS-mediated apoptosis (Tian et al. 1995; Izquierdo and Valcárcel 2007). The observed copy loss might have facilitated escape of self-reactive lymphocytes from proliferation and apoptosis checkpoints.

Four of the six mice with the memory-only sorting profile (M20, M21, M22, and M24) have a comparable number of CNAs to the other 19 samples (M1–M19), but M23 and M25 had a very large number of CNAs, most of which were recurrent loci between the two mice. Chr X and Y probes clearly showed a male and female genome, ruling out possible sample duplication through a labeling error (Supplemental Table S2). The high numbers of CNAs in these two mice is reminiscent of CNAs observed in a malignant genome of a leukemic cell and involve many genes, some of them of known immune function. Three examples of these CNAs include a recurrent CNA in M23 and M25 that is a copy gain at Chr 9: 20,856,727–21,236,076 spanning several genes, including *Keap1, S1pr5,* and *Ilf3* with hematopoietic cells expression and an immune-related function (Table 2; Supplemental Fig. S5A; Wu et al. 2016). Another large CNA is at Chr 10: 79,084,598–81,041,680. This recurrent CNA spans a large number of genes of which several also have a high or exclusive expression in hematopoietic cells, including *Madcam1, Gzmm, Rps15, Mknk2, Dapk3, Matk, S1pr4,* and *Abca7* (Table 2; Supplemental Fig. S5B; Wu et al. 2016). Another is a recurrent copy gain CNA in these two mice also at Chr 10: 127,684,108–128,334,229, also spanning several genes with high or exclusive hematopoietic cell expression and/or an immune function, including *Il23a, Pa2g4, Dgka*, and *Coq10a* that are expressed in lymphocytes (*Dgka* is exclusively expressed in CD4^+^ and CD8^+^ cells) (Table 2; Supplemental Fig. S5C).

**Table 2.**
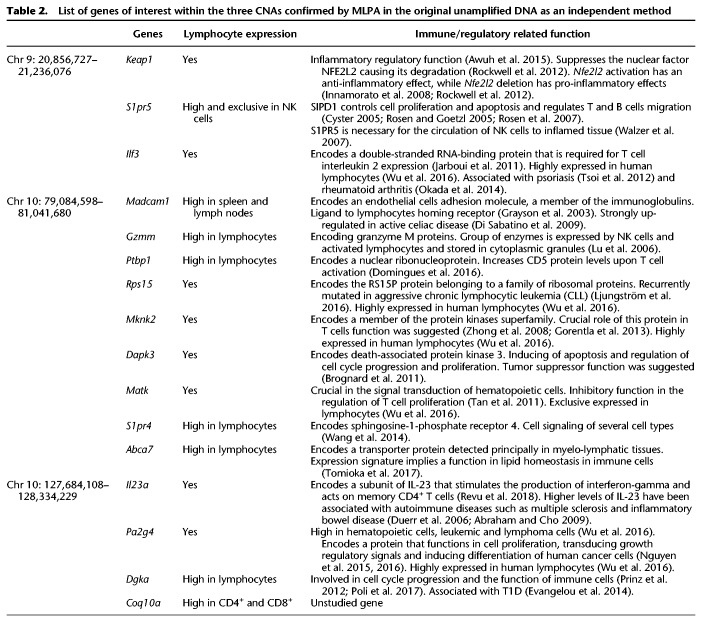
List of genes of interest within the three CNAs confirmed by MLPA in the original unamplified DNA as an independent method

**Figure 5.**
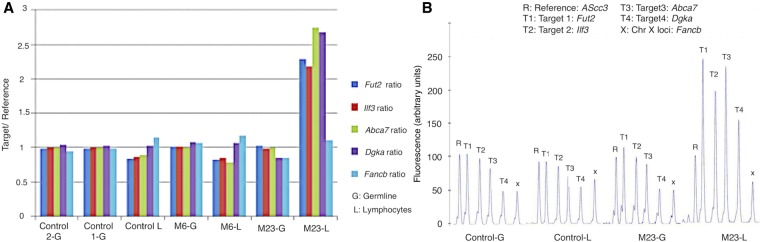
Independent confirmation of CNAs in original unamplified DNA by MLPA. Four recurrent loci with copy gain in M23 and M25 spanning genes with immune function (Supplemental Fig. S5) were tested, along with DNA from three mice that did not have the CNA (M6-L and -G, control L and 1-G, and control 2-G). (*A*) The height of the MLPA peak at each locus for each mouse is shown in proportion to the reference probe. Only the four loci in the T cell (but not the tail representing the germline) DNA from mouse M23 show an increase over 1. (*B*) Representative peaks shown as a percentage of the reference probe. Only mouse M23 shows doubling of the peak in T cells.

Since the above recurrent CNAs span several genes with a high lymphocyte expression and have an immune and proliferation function in immune cells (Table 2), we aimed to confirm these CNAs in the original unamplified DNA by multiplex ligation-dependent probe amplification (MLPA), the gold standard method for CNV diagnostics (Feuk et al. 2006) as independent validation. We selected three genes within the above three recurrent CNAs, that are expressed in lymphocytes and have an immune- and/or proliferation-related function and in which we have enough of the original unamplified DNA to be examined by MLPA: *Ilf3, Abca7*, and *Dgka*. *Ilf3* on Chr 9 is highly expressed in lymphocytes and encodes a double-stranded RNA-binding protein that is required for T cell interleukin 2 expression (Jarboui et al. 2011). Moreover, it is associated with two immune-related diseases: psoriasis (Tsoi et al. 2012) and rheumatoid arthritis (Okada et al. 2014). *Abca7* on Chr 10 encodes a transporter protein detected principally in myelo-lymphatic tissues with an expression signature that implies a function in lipid homeostasis in immune cells (Tomioka et al. 2017). *Dgka* is exclusively expressed in CD4^+^ and CD8^+^ cells, and its encoded protein is involved in cell cycle progression and the function of immune cells (Prinz et al. 2012; Poli et al. 2017) and has been reported to be associated with T1D (Evangelou et al. 2014). We successfully confirmed by MLPA the above three CNAs (Fig. 5A,B). Further detailed annotation of the CNAs seen exclusively in M23 and M25 was not attempted because of their large number and the likelihood that they are a consequence rather than a cause of the rapid proliferation.

### T cells involved in host defense have smaller copy number changes

As a normal host-defense control, we tested DNA from memory CD4^+^ T cells from the popliteal lymph nodes of 10 NOD mice (eight females and two males; 9–14 wk) infected with *Leishmania major* (*L. major)*. Out of 10, two had CNAs (one had one and the other one had two CNAs) (Supplemental Table S3; Supplemental Data S4). The same experiment was done on seven non-autoimmunity susceptible BALB/c mice (19 wk) to determine if the occurrence of CNAs in lymphocytes is promoted by an autoimmune genetic background. Out of the seven mice, three had CNAs (1–2 CNAs) (Supplemental Table S3; Supplemental Data S4). The mean size of CNAs in autoreactive cells of diabetic mice (26.02 ± 1.69, mean ± SEM, *n* = 403) was significantly twofold larger than that in samples from *L. major*-infected mice (7.71 ± 2.03, *n* = 7; Fisher-Pitman permutation test, *P* = 0.0019).

### The CNAs occur in prethymic precursors

Sequencing the TCR of the CD4^+^ memory cells from PLN of 10 diabetic mice showed low clonality in both the α-chain and β-chain (Supplemental Data S5), too low to explain the level of mosaicism found in the CNAs, suggesting prethymic occurrence in a marrow progenitor. We found that the most common TCR is (CDR3-V-J: CVVGDRGSALGRLHF-TRAV11*01-TRAJ18*01) sequence for the α-chain and (CDR3-V-J-D: CAASSNTDKVVF-TRBV2*01-TRBJ1-1*01-TRBD1*01) sequence for the β-chain are present within all samples with relatively high frequency.

## Discussion

This study demonstrates a novel discovery of somatic copy-number mutations in immune-related genes in memory cells of diabetic NOD mice, an excellent model for human T1D. Some of these genes are highly expressed in T cells and may well represent an important causal contribution in the autoimmunity of the NOD model and, quite plausibly, of human T1D. Their causal role is supported by the nonrandom recurrence of eight of these PZMs affecting the same genes in different mice. In the paradigm of cancer, independent recurrence of somatic mutations, either point or copy-number, in different patients is considered strong evidence that the mutation is causal. In fact, this is the criterion by which most tumor-suppressor genes have been identified. Somatic mutations are a common occurrence, but, being confined to one single cell and its progeny, they are unlikely to cause disease, unless the cell progeny expands exponentially, as is the case in cancer (Jacobs et al. 2012; Li et al. 2014) and, to a lesser extent, in autoreactive cells.

We considered alternative explanations for our observations and found them unlikely. Hyperglycemia could accelerate the normal accumulation of somatic mutations in blood cells, as described for type 2 diabetes (Bonnefond et al. 2013; Loftfield et al. 2018), but the alterations we observed were much more drastic, over a much shorter timeframe (euthanasia within days of hyperglycemia onset). It is possible that some of our findings are DNA amplification artifacts. Against it is the control experiments described under Methods and the MLPA confirmation using unamplified DNA, especially in mice M23 and M25, who had the highest number of CNAs, i.e., the most likely to be due to technical artifacts. Also, in our mixture experiments, we found the same loci in all three mixes, or in the two highest ones, always at the same locus. This is very unlikely to happen at random, if these were amplification artifacts. Overlaps also are against artifacts, which should have happened at random.

The contribution of somatic mutations in the pathogenesis of cancer has been well established. A PZM in a tumor suppressor gene in a single cell causes a monoclonal expansion during which additional PZMs occur, causing more pathological phenotypes and further release from proliferation controls (Watson et al. 2013). The identification of such driver mutations in cancer has enabled therapeutic advances by identifying drug targets. One example is Gleevec, a selective inhibitor of the BCR-ABL tyrosine kinase, the pathogenic fusion transcript in chronic myeloid leukemia (CML) (Hernández-Boluda and Cervantes 2002).

In the case of autoimmune diseases, similar to cancer, disease is caused by a proliferation of clonal lymphocytes from a small number of autoreactive cells that have escaped natural tolerance to self-antigens. Although autoimmunity involves more functional steps than proliferation and resistance to apoptosis, it does share these two features with cancer. Natural tolerance involves many checkpoints. Its failure is likely a multistep process, causing autoimmune diseases to develop over many years. It has been estimated that as many as 50% of all T cell receptor V(D)J rearrangements will recognize some autoantigen (Ignatowicz et al. 1996). Most of these will be deleted by negative selection in the thymus, where tissue-specific antigens like insulin are expressed (Vafiadis et al. 1997; Liston et al. 2004). However, some autoreactive T cells escape to the periphery and must be dealt with by peripheral tolerance. A large number of molecular players are involved in each of these checkpoints (for review, see Goodnow et al. 2005), and many others may be important but still unstudied.

Epidemiological evidence has connected autoimmunity to hematologic malignancy in several scenarios. The risk of lymphoid malignancy is higher in individuals affected by many autoimmune conditions, such as celiac disease, lupus, rheumatoid arthritis, and Sjorgen's syndrome (Goodnow 2007). Somatic *FAS* mutations in non-Hodgkin's lymphoma are often seen in patients with a prior history of autoimmunity (Grønbæk et al. 1998). This suggests overlaps between immune tolerance checkpoints, important in autoimmunity, and proliferation checkpoints, important in lymphoid malignancy.

The plausibility of this model depends on how often such PZMs happen in autoimmunity-relevant hematopoietic lineages. PZMs originated in hematopoietic stem cells and progenitor cells have been identified in peripheral whole blood (Busque et al. 2012; Genovese et al. 2014; Xie et al. 2014; Young et al. 2016). SNP-array data show that whole-blood DNA has mosaic, Mb-sized CNAs in about 5% of healthy individuals (Forsberg et al. 2012; Jacobs et al. 2012; Laurie et al. 2012). This detectable mosaicism is due to a proliferative or survival advantage of the copy-number mutation (versus early occurrence in embryogenesis), as it is age-dependent and variable over time (Forsberg et al. 2012). Only a very small fraction of all people carrying them will develop leukemia, leaving a broad gray zone for a role in nonmalignant disease. In a related study of SNP-array discrepancies between monozygotic twins, we calculated an average, ∼130 point-PZMs per individual, at high enough mosaicism, in whole blood, to flip a SNP genotype call (Li et al. 2014). Single-cell sequencing has more recently revealed that somatic mutations are common and accumulate in normal cells (Abyzov et al. 2012; McConnell et al. 2013; Lodato et al. 2015, 2018; Blokzijl et al. 2016; Bae et al. 2018).

In logarithmically expanded autoantigen-specific lineages, mosaicism might be much higher and still not detectable without isolating these lineages from the rest of the blood cells. A recent publication identified an exclusive somatic mutation in the expanded CD8^+^ memory subset in newly diagnosed rheumatoid arthritis (Savola et al. 2017).

In six of the 25 mice, all cells in PLNs were memory, and two of these mice showed a very unstable genome with many recurrent CNAs. This instability might be explained by the copy gain in two histone loci (Fig. 4C). Histone overexpression due to copy gain is very likely to have increased the normally tightly regulated expression of these nucleosomal proteins, whose correct expression upon DNA replication is crucial to genomic integrity (Mejlvang et al. 2014). The multiple nearly identical genes encoding these two histones also make the locus susceptible to rearrangements. The remaining CNAs cover a large number of RefSeq genes, most of which are not functionally annotated and have no published literature.

Activated T cells in the context of host defense with *L. major* also have CNAs, but they are fewer (seven in 17 mice versus 403 in 25 mice) and significantly smaller than those observed in T cells involved in NOD autoimmunity. Moreover, their occurrence in BALB/c indicates that they are not due to the germline determinants of autoimmunity.

Identification of the genes affected by the CNAs in the autoreactive lymphocytes could serve as a potential therapeutic target as their CNAs exist only in disease-causing cells. One example of these is recurrent CNAs that span *Igflr1* (Supplemental Data S3) and *Tmem107* (Fig. 4A) encoding transmembrane proteins highly expressed in T cells. Their amplification or deletion should be detectable by flow cytometry in blood cells, providing a potential biomarker and therapeutic target.

Sequencing the TCR of 10 diabetic mice revealed a percentage of clonotypes too low to explain the observed levels of mosaicism, suggesting that the CNAs are occurring prior to the thymic selection in a bone marrow progenitor and not in the antigen-specific lineage. Additional PZMs, occurring during the autoantigen-activated logarithmic expansion of T cells likely to happen but their level of mosaicism would have been too low to detect by our approach, as we did not isolate cells by antigen specificity.

It is possible that the overlap could be due to a mechanistic origin due to the fact that some parts of the genome are more fragile than others. However, there is a very large number of such loci, making their recurrence unlikely. Moreover, none of our findings correspond to known CNVs between mouse strains.

Since these postzygotic mutations do not have to pass selection for fitness of the whole organism, they are likely to have more drastic functional effects than germline variants. If so, they could be ideal therapeutic targets, as they exist only in disease-causing cells. These data must be confirmed and expanded using CNA detection in single cells by sequencing of multiple cell clones that will enable a more accurate detection and estimate of the level of genetic mosaicism (Abyzov et al. 2012; McConnell et al. 2013; Lodato et al. 2015; Blokzijl et al. 2016; Bae et al. 2018; Lodato et al. 2018). Future studies should address the causal role by gene editing and extend the studies to human T1D autoimmunity. Other autoimmune diseases should be likewise examined and detection of point mutations by exome sequencing is planned, to explore the full PZM spectrum.

## Methods

### Study approval

In this study, all experiments on animals were approved by the Facility Animal Care Committee (FACC) according to the guidelines established by the Canadian Council on Animal Care and the McGill University Animal Care Committee (AUP#7517).

### Experimental model

Animals were obtained from standard sources and the diagnosis of diabetes, euthanasia, and dissection were carried out by standard precedures (Supplemental Methods).

### Isolation of memory T lymphocytes

Nodes were placed in 1 mL cold Dulbecco's saline DPBS (1×) (cat#: 14190-144, Life Technologies) and lymphocytes isolated by sheer-force slide dissociation. Next, fluorescence activated cell sorting (FACS) was used to isolate memory T lymphocytes (CD4^+^/SELL^−^/CD44^+^ by a FACSAria cell sorter (Becton-Dickinson). In these experiments, cells were stained using: anti-CD4^+^-FITC (cat#: 11-0042-81, eBioscience), anti-CD8α^+^-PE (cat#: 12-0081-82, eBioscience), anti-CD62L^+^-APC (naive-cell marker; cat#: 130-091-805, Miltenyi Biotec), and anti-Human/Mouse CD44^+^-PreCP-cy5.5 (memory-cell marker; cat#: 45-0441-82, eBioscience). All samples were kept on ice throughout isolation and staining.

### Whole-genome amplification

DNA was amplified using a GenomePlex complete whole-genome amplification (WGA-2) (cat#: WGA2-50RXN) kit, specifically approved by Agilent for aCGH to detect copy-number differences. DNA was next purified using a PCR Cleanup kit from Sigma-Aldrich (cat#: NA1020).

### Detection of PZMs

We sent 1.5 µg of amplified DNA to Oxford Gene Technologies (OGT) for comparative genomic hybridization analysis on the mouse 4 × 180 K Agilent array. DNA from memory cells was the test sample, with germline DNA from the tail as reference. To model the sensitivity to detect levels of mosaicism, germline NOD DNA was mixed with C57BL6/J DNA prior to amplification. The NOD proportions were 100%, 50%, and 25%, to imitate levels of mosaicism at inter-strain copy-number variants (CNV). Reference was 100% C57BL6/J.

Copy-number alterations (CNAs) were detected by the Agilent dual color, 4 × 180 k mouse CGH array (Supplemental Methods). CGH quality control (QC)- derivative log ratio spread (DLRS) values were within Agilent Technologies specifications (Supplemental Fig. S2).

To evaluate the specificity of the method, two technical controls were tested in which DNA from lymphocytes from C57BL6/J mouse was amplified twice by the same kit (WGA-2), and then one of the two amplified samples was used as test and the other as a reference in CGH (4 × 180 K) analysis. Moreover, DNA was amplified twice from memory CD4^+^ T cells obtained from PLN of a diabetic mouse (M9) and twice from germline DNA from a tail-clip sample of the same mouse. These duplicate samples were analyzed separately (M9 and M9B) by CGH using in both samples the amplified memory CD4^+^ DNA as test and the amplified germline DNA as reference.

### Bioinformatics analysis

Test and reference signals were calculated for each probe and internal normalization equalized the systematic difference between the two fluorochromes. The base-2 log of the Cy3/Cy5 ratio (LRR) was plotted against chromosomal coordinates. Probes covering NOD or BALB/c germline deletions were eliminated. Regions bearing copy-number aberrations were identified using two independent algorithms, Agilent Genomic Workbench 7.0 (Agilent Technologies) (Roy and Motsinger Reif 2013) implementing ADM-2 algorithm and DNAcopy software implementing the circular binary segmentation (CBS) algorithm (Venkatraman and Olshen 2007). In ADM-2 (threshold 6.0), we applied Agilent Technologies recommended filtering standards of a minimum of three aberrant consecutive probes. The mouse genome UCSC mm9 NCBI build 37/July 2007 was used to match the indexing of the Agilent probes (converting coordinates did not affect the findings).

CNAs were kept as true if called by both algorithms ADM-2 (LRR ≥ |0.25|, *P*-value ≤ 2.90 × 10^−07^, Bonferroni threshold) and CBS (LRR|0.25|, *P*-value ≤ 1 × 10^−04^). The lower statistical significance threshold for CBS is justified by the vastly smaller number of hypotheses tested (number of ADM-2-called CNAs).

Tissue-specificity of the expression levels of the genes involved was obtained from mouse and human BioGPS data, 2018 (Wu et al. 2016).

### Statistics

The statistical significance of the recurrence of overlapping CNAs in different mice was estimated by permutation testing (Supplemental Methods). Either the actual length in kilobases of each CNA or the number of probes covering it were used in this simulation. Both gave similar results, but we retained the results of the probe permutation, a more conservative approach that avoids inflating the statistical significance because of nonhomogenous distribution of the probes (favoring coding genes).

The difference in the number of probes covering CNAs in T cells from diabetic pancreatic nodes versus *L. major*-infected popliteal nodes was assessed by the Fisher-Pitman permutation test, which does not require an assumption about normality of distribution. We compared the number of probes (number of consecutive probes covered); because of the gene-centered positioning of the probes, they better reflect the genes covered by them than the actual CNA length.

### Multiplex ligation-dependent probe amplification

Some CNAs were selected for confirmation by MLPA on unamplified DNA by a standard protocol (Supplemental Methods). Primer and probes used for the MLPA experiment can be found in Supplemental Table S4.

### TCR sequencing

RNA samples were extracted from the CD4^+^ memory cells isolated from the PLN of diabetic mice and amplified by a 5′ RACE approach using the SMARTer RACE cDNA amplification kit by Clontech and sequenced on an Illumina MiSeq apparatus (Supplemental Methods).

## Data access

All raw and processed CGH data generated in this study have been submitted to the NCBI Gene Expression Omnibus (GEO; https://www.ncbi.nlm.nih.gov/geo/) under accession number GSE114660. All raw and processed sequencing data generated in this study have been submitted to the NCBI Sequence Read Archive (SRA; https://www.ncbi.nlm.nih.gov/sra/) under accession number SRP148447.

## Supplementary Material

Supplemental Material
