# Supplementary material for: Clonal copy-number mosaicism in autoreactive T lymphocytes in diabetic NOD mice
Source: Genome Res. 2019 Dec;29(12):1951–61. doi: 10.1101/gr.247882.118 (PMC6886509; doi:10.1101/gr.247882.118)
Supplement: Supplemental Material [file supp_29_12_1951__index.html]

Supplemental Material 

# Clonal copy-number mosaicism in autoreactive T lymphocytes in diabetic NOD mice

## Supplemental Material

- Supplemental\_Data\_S1.xlsx
- Supplemental\_Data\_S2.xlsx
- Supplemental\_Data\_S3.xlsx
- Supplemental\_Data\_S4.xlsx
- Supplemental\_Data\_S5.xlsx
- Supplemental\_Materials.docx
