# Supplementary material for: Clonal copy-number mosaicism in autoreactive T lymphocytes in diabetic NOD mice
Source: Genome Res. 2019 Dec;29(12):1951–61. doi: 10.1101/gr.247882.118 (PMC6886509; doi:10.1101/gr.247882.118)
Supplement: Supplemental Material [file supp_gr.247882.118_Supplemental_Materials_.docx]

Supplemental Materials for: Clonal copy-number mosaicism in autoreactive T-lymphocytes in diabetic NOD mice

Maha Alriyami^1, 2^; Luc Marchand^1^; Quan Li^1, 3^; Xiaoyu Du^1^; Martin Olivier^4^; Constantin Polychronakos^1*^

^1^The Endocrine Genetics Laboratory, Child Health and Human Development Program and Department of Pediatrics, McGill University Health Centre Research Institute, Montreal, Quebec, Canada, H3H 1P3. ^2^Department of Biochemistry, College of Medicine and health Sciences, Sultan Qaboos University, 35, Al-Khoud, 123, Muscat, Oman. ^3^University of Toronto, Princess Margaret Cancer Centre. ^4^Departments of Medicine, Microbiology and Immunology, McGill University Health Centre Research Institute, Montreal, Quebec, Canada, H3H 1P3.

Correspondence and requests for materials should be addressed to Constantin Polychronakos: The Endocrine Genetics Laboratory, Child Health and Human Development Program and Department of Pediatrics, McGill University Health Centre Research Institute, Montreal, Quebec, Canada, H3H 1P3, email: [constantin.polychronakos@mcgill.ca](mailto:constantin.polychronakos@mcgill.ca), Phone #: 514-412- 4315

**Electronic supplementary material**

**Supplementary figures and tables**

**This PDF file includes:**

Supplemental Methods

Figs. S1 to S5

Table S1 to Table S4

**Table of contents**

| **Supplemental Methods**………………………………………………………………………...……. | 2 |
| --- | --- |
| **Fig. S1.** Survival curve of NOD mice showing the percentage of deaths per week for Female and Male mice…..……………………………………………………………………………………..…... | 5 |
| **Fig. S2.** Derivative log ratio spread (DLRS)-Quality control values for tested samples using Comparative Genomic Hybridization analysis (CGH) on the mouse 4X180K Agilent array.……….. | 6 |
| **Fig. S3.** CGH array results showing recurrent CNAs in memory cells from PLNs of diabetic mice... | 7 |
| **Fig. S4.** Additional interesting recurrent CNAs in two mice or more………………………………... | 8 |
| **Fig. S5.** Independent confirmation of CNAs in original unamplified DNA by MLPA…………….... | 9 |
| **Table S1.** Number of CNAs found in memory CD4^+^ lymphocytes from PNLs of tested mice……… | 10 |
| **Table S2.** The average of all probes of mice M23 and M25 in each chromosome clearly shows the sex differences between them.………………………………………………………………………. | 11 |
| **Table S3.** Number CNAs found in memory CD4^+^ T cells from the popliteal lymph nodes of 10 NOD mice and 7 BALB/c mice infected with *L. major*…...……………………………………….… | 12 |
| **Table S4.** Primer and Probes used for MLPA experiment…………………………...………………. | 13 |

**Supplemental Methods**

**Animal Model**

Similar to human T1D, NOD diabetes is caused by a combination of polygenic inheritance and environmental factors ([Pearson et al. 2016](#_ENREF_56); [Polychronakos & Li 2011](#_ENREF_58)). Female mice are predominantly affected (90- 100% by 30 weeks of age) compared to males that develop it at an older age with lower frequency.

Mice were kept in pathogen-free conditions (maximum of five mice per cage) and were fed a standard chow diet (Tekland irradiated global 18% protein rodent diet, 2918). During the study, animals were housed with lights on from 7:00 am to 7:00 pm, and access to food and water as ad libitum. Wild-type NOD/ShiLtJ (purchased from The Jackson Laboratory, Bar Harbor, ME) and BALB/c (purchased from Jackson Laboratory and Charles River) mice were monitored for glycosuria every two days. The onset of diabetes was detected by making the animal urinate on a reagent strip, ChemStrip uG/K (cat#: 10647705119, Roche), to detect glucose. Positive animals for glycosuria (>13.8 mmol/l (250 mg/dl) were euthanized and blood glucose was measured using a glucometer. All animals that fit the inclusion criteria were used (n= 25, 22 females and three males, Supplemental Table S1); there were no exclusion criteria and no randomization were applied. Since we had no prior information on which to base power calculations, the number of mice tested was determined by available funds. Diabetic mice were euthanized with CO2 and dissected to obtain pancreatic lymph nodes (PLN) for the isolation of memory CD4^+^ T cells. Tail samples were also obtained for sampling germline. For four mice, cells from other peripheral lymph nodes (Superficial cervical LN, Axillary LN and Inguinal LN) were also obtained as controls.

To compare with T cells proliferating in normal host defense, ten NOD (n= 10, Two males and eight females) and seven BALB/c mice (n= 7, females) (Supplemental Table S3) were infected with *Leishmania major* by injecting 5×106 late stationary phase *L. major* promastigotes (50 μl PBS) subcutaneously into the footpad. Three weeks after injection, mice were euthanized, and the popliteal lymph node was dissected. Blinding between diabetes and *L. major* infected animals was not possible because of the different source of material. However, the array data generation and the bioinformatics analysis were done blindly in one batch, using the same parameters.

**Detection of PZMs**

Test and reference DNA samples were labeled with different fluorescent tags (Cy3 and Cy5, respectively), which were next hybridized to the probes of the 4×180 chip, arrayed genome-wide but with concentration in and around coding sequences. The fluorescence intensity ratio (Cy3: Cy5) at each probe position reflects copy-number differences between the two DNA samples. In CGH if test and reference DNA are equal, the LRR is zero (log of the ratio 2 copies/ 2 copies = 0), which plotted against the chromosomal coordinates in bp, display a small amount of noise around 0. Series of consecutive probes drastically deviating from background noise indicates a copy-number change.

**Statistics**

The statistical significance of the recurrence of overlapping CNAs in different mice was estimated by permutation testing. The chromosomal coordinates of each CNA were randomly reassigned around the mouse genome and the number of overlaps noted. This process was repeated for 100,000 iterations. Either the actual length in kb of each CNA or the number of probes covering it were used in this simulation. Both gave similar results, but we retained the results of the probe permutation, a more conservative approach that avoids inflating the statistical significance because of non- homogenous distribution of the probes (favoring coding genes).

**Multiplex Ligation-dependent Probe Amplification (MLPA)**

Some CNAs were selected for confirmation by MLPA based on the presence of genes with an immune function and a lymphocytes expression. MLPA on the original unamplified DNA samples was used as the gold standard in CNV diagnostics. Primers (Supplemental Table S4) were designed for four loci. The amplification was carried out by PCR using 33 cycles, of 95°C for 30 seconds, 60°C for 30 seconds and 72°C for 1 minute. Amplification products were identified and quantified by capillary electrophoresis on an ABI310 genome analyzer. The peak areas of the PCR products were determined by GeneMapper software. Data were normalized by dividing each probe’s peak area by the average peak area of the control gene in each sample. The normalized peak pattern was divided by the average normalized peak pattern of all the samples in the same experiment.

TCR sequencing

PCR products were purified using the QIAquick PCR purification kit by Qiagen and prepared for MiSeq sequencing with the *Illumina* Nextera XT library preparation kit according to the manufacturer's manual. Sequencing was done using the MiSeq paired-ends (250 bp) protocol. The reads were aligned to the TCR reference sequence of the international ImMunoGeneTics information system (IMGT). The alignment assigned V and J segments in both the α and β chain. The CDR3 (non-template added random amino acids) was determined starting from the V segment conserved Cysteine (Cys) at position 104 and the conserved Phenylalanine/Tryptophan (Phe/Trp) at positions 118 in the J segment.


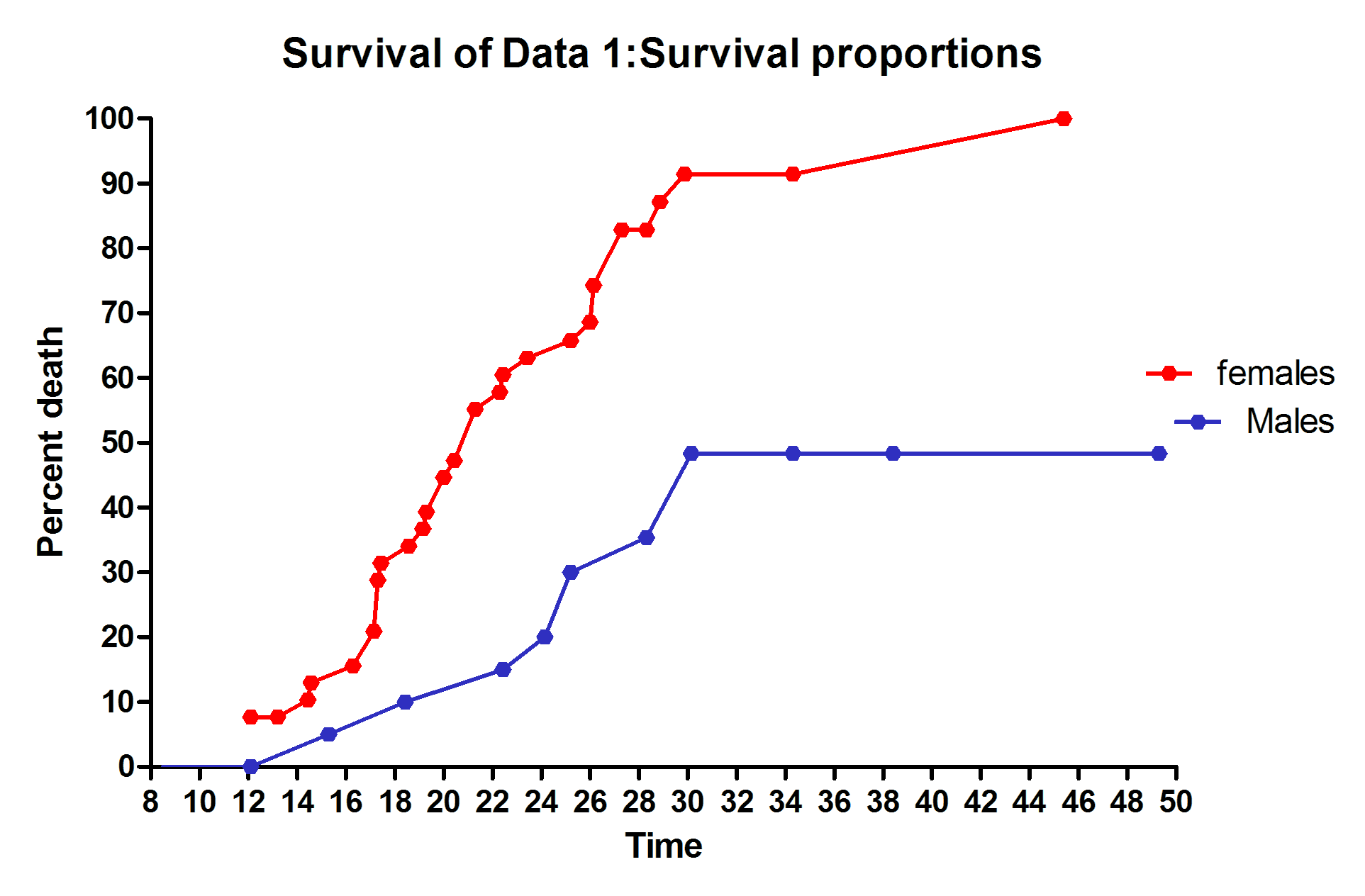


**Fig. S1.** Survival curve of NOD mice showing the percentage of deaths per week for Female and Male mice.


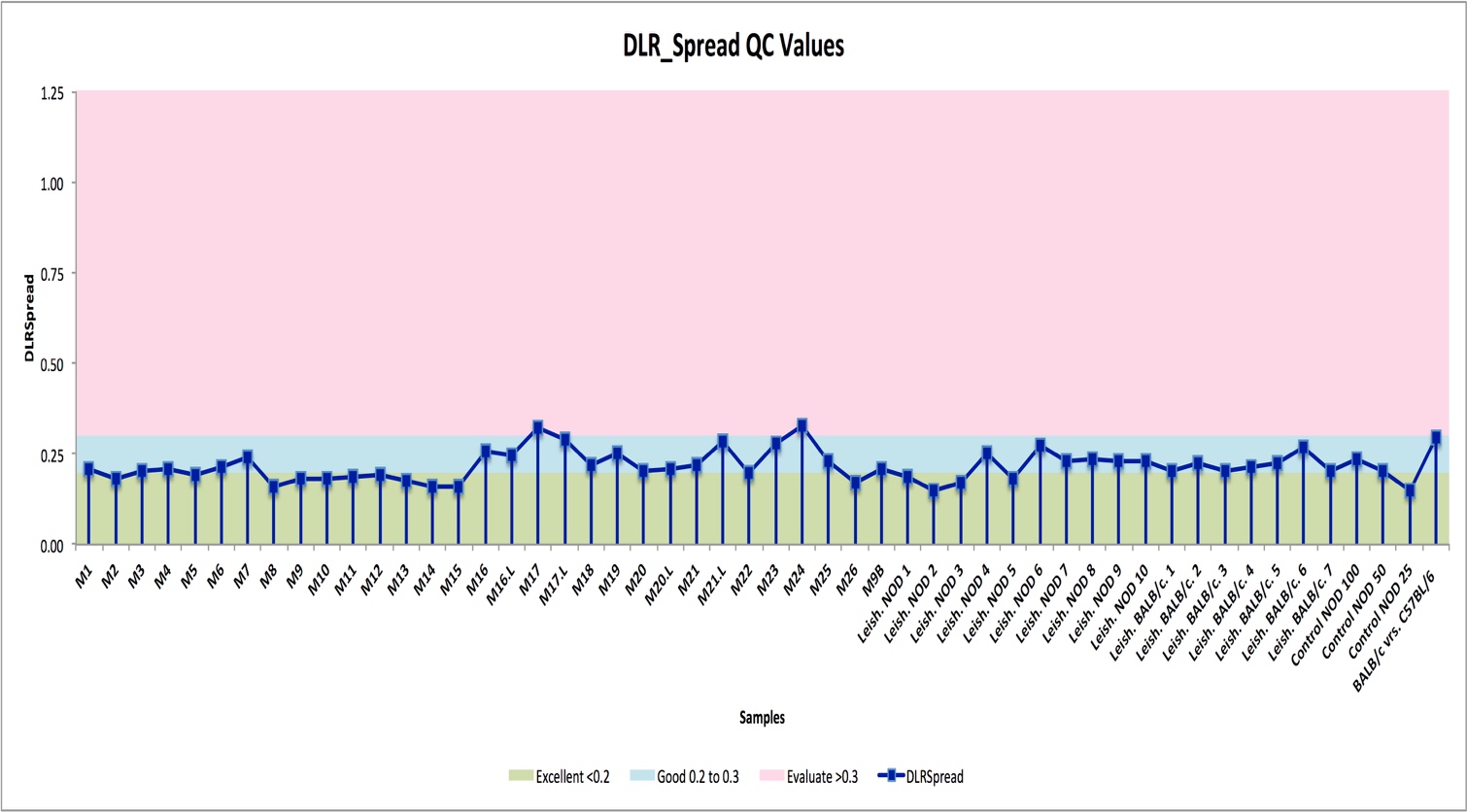


**Fig. S2.** Derivative log ratio spread (DLRS)-Quality control values for tested samples using Comparative Genomic Hybridization analysis (CGH) on the mouse 4X180K Agilent array. DLRSpread values are within Agilent technologies specifications.

**
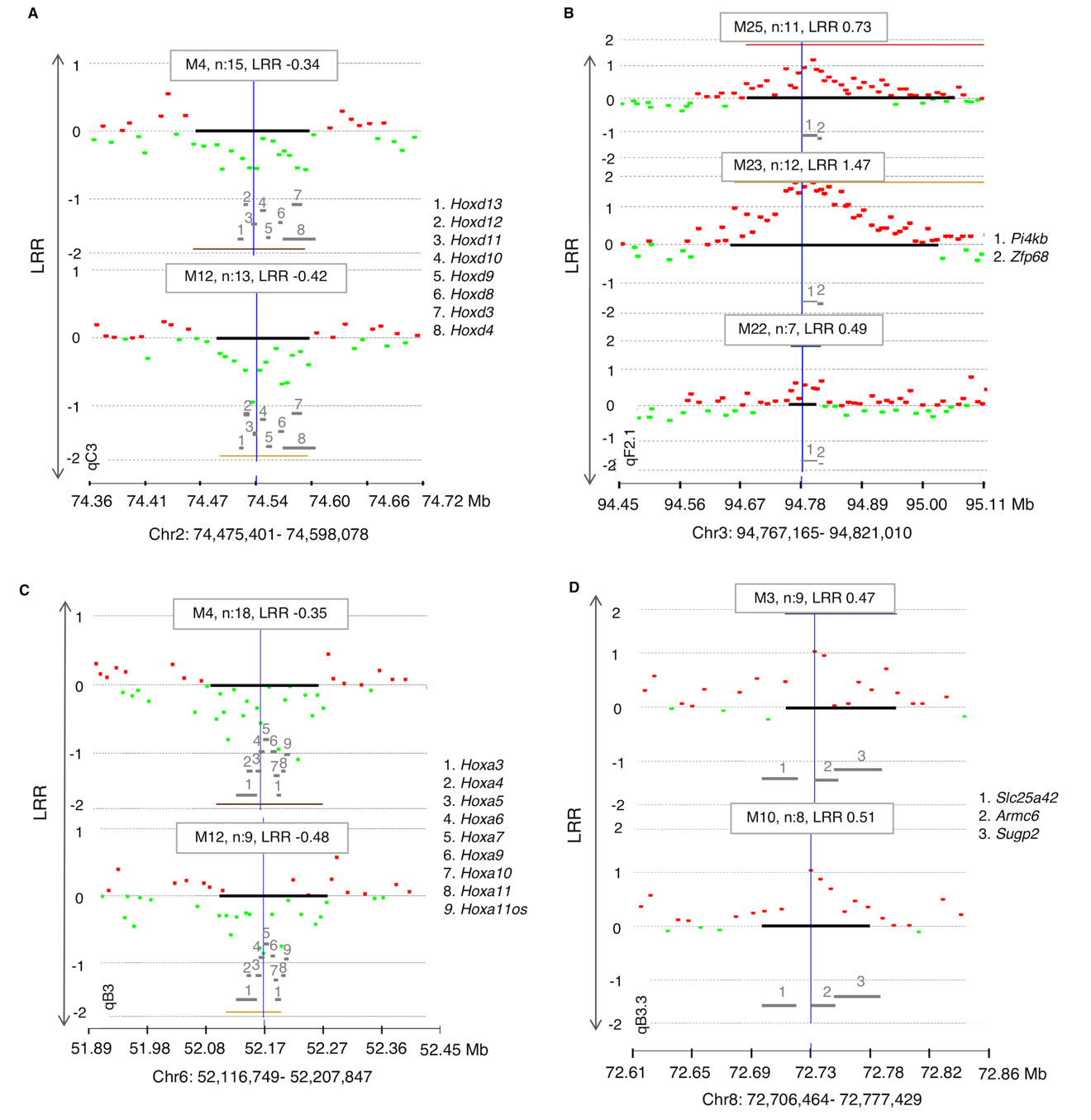
**

**Fig. S3.** CGH array results showing recurrent CNAs in memory cells from PLNs of diabetic mice (n: number of affected probes, location of genes is indicated by numbers). (*A* and *C*) Two recurrent copy losses spanning *Hoxa* and *Hoxd* gene family. (*B*) A recurrent copy gain in cells of three mice spanning *Pi4kb*. (*D*) A recurrent copy gain in two mice spanning *Armc6*.


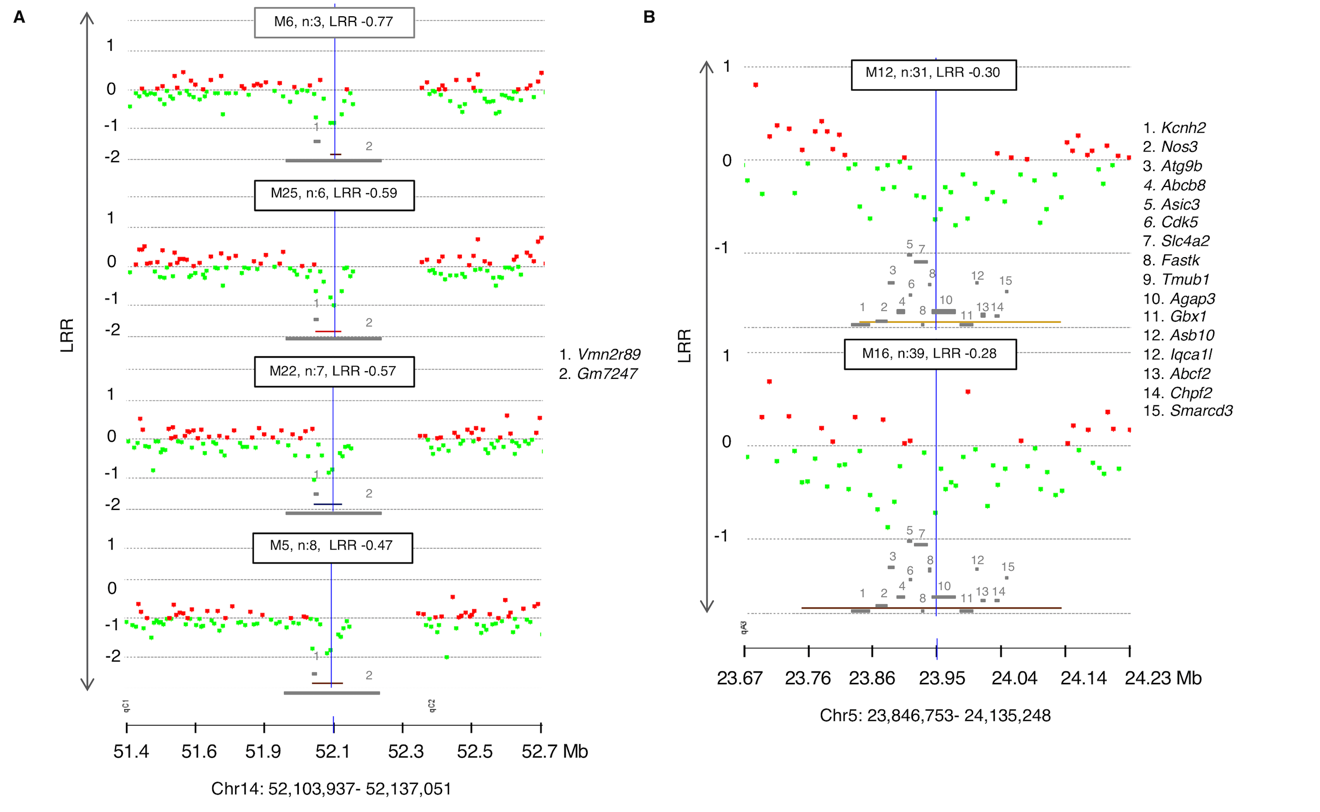


**Fig. S4.** Additional interesting recurrent CNAs in two mice or more. **(*A*)** CNA is in chr14 (chr14: 52,061,318- 52,145,237) in the memory cells of M5 and M22 is a copy loss that spans *Gm7247* and *Vmn2r89.* These two genes are also spanned by copy losses CNAs in mice M6 and M25 called by ADM2, however, missed the CBS cut off. (***B)*** A recurrent CNA is a copy loss in chromosome 5 (chr5: 23,846,753- 24,135,248) found independently in two different diabetic mice M16 and M12 in which both were called by ADM2, however, missed the CBS cut off for one of the mice (M16).

**
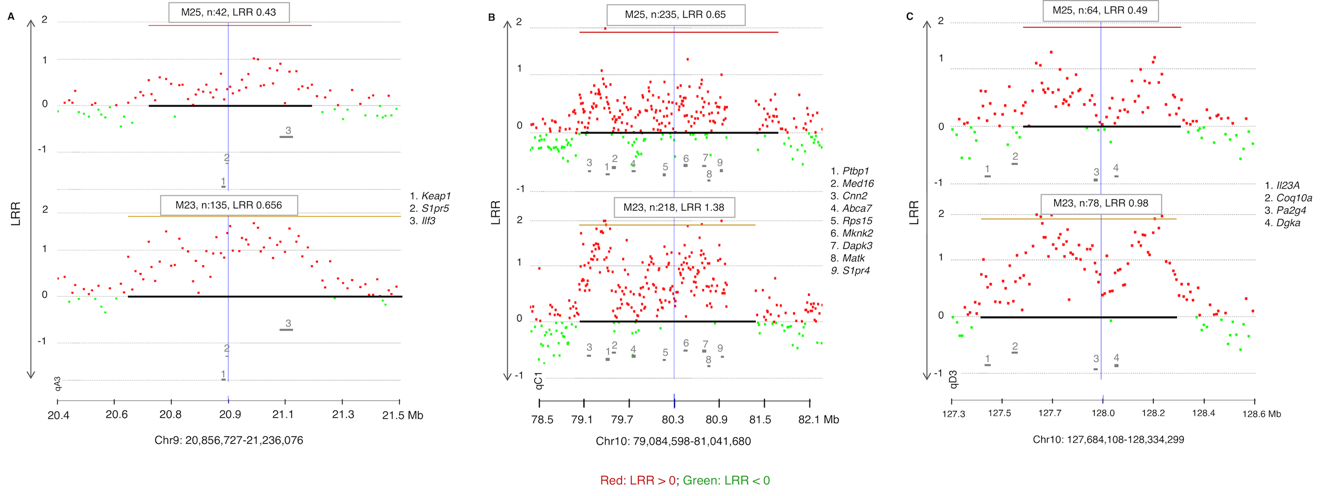
**

**Fig. S5.** Independent confirmation of CNAs in original unamplified DNA by MLPA. Four recurrent loci with copy-gain in M23 and M25 spanning genes with immune function were tested, along with DNA from two other mice that did not have the CNA (n: number of affected probes). *A*, *B* and *C* show CGH results at three different loci (DLRSpread M23: 0.28, M25: 0.23).

**Table S1.** Number of CNAs found in memory CD4^+^ lymphocytes from PNLs of tested mice (DLRSpread values; Supplemental Fig. S2).

| **Mouse Sample** | **Sex F/M** | **Age (weeks)^a^** | **Blood Glucose^b^** | **#CNAs** |
| --- | --- | --- | --- | --- |
| M1 | F | 18.4 | 11.1 | 0 |
| M2 | F | 9 | 13.7 | 0 |
| M3 | F | 18.4 | 15.8 | 1 |
| M4 | F | 23.1 | 16.3 | 3 |
| M5 | F | 19 | 18.9 | 1 |
| M6 | F | 27.3 | 19.5 | 0 |
| M7 | F | 22.6 | 20.5 | 1 |
| M8 | F | 15.1 | 25.6 | 0 |
| M9 | F | 20.3 | 26.2 | 0 |
| *M9B ^c^* | F | 20.3 | 26.2 | 0 |
| M10 | F | 23.7 | 27 | 1 |
| M11 | F | 19 | 30.5 | 0 |
| M12 | F | 14.1 | High | 4 |
| M13 | F | 17.3 | High | 0 |
| M14 | F | 18.7 | High | 0 |
| M15 | F | 23.7 | High | 0 |
| M16 | F | 24.3 | 19.3 | 1 |
| *M16L^d^* | F | 24.3 | 19.3 | 0 |
| M17 | F | 20.1 | High | 0 |
| *M17L^d^* | F | 20.1 | High | 0 |
| M18 | M | 22.3 | High | 1 |
| M19 | M | 24.1 | 22.2 | 0 |
| M20 | F | 21.2 | 22.5 | 0 |
| *M20L^d^* | F | 21.2 | 22.5 | 0 |
| M21 | F | 29.1 | 15.8 | 0 |
| *M21L^d^* | F | 29.1 | 15.8 | 0 |
| M22 | F | 23.6 | 19.9 | 5 |
| M23 | F | 14 | 25.1 | 295 |
| M24 | F | 21.3 | 20.7 | 0 |
| M25 | M | 18.4 | 22.7 | 90 |

^a^ Age: Age of disease onset in weeks.

^b^ Blood Glucose in mmol/L

^c^ M9B a duplicate amplification of sample M9

^d^ M16L, M17L, M20L and M21L: samples from the peripheral LNs from the same mice M16, M17, M20 and M21 respectively.

**Table S2.** The average of all probes of mice M23 and M25 in each chromosome clearly shows the sex differences between them. Fluorescent signals for test and reference intensity is in normalized arbitrary units.

| **M23** | | | **M25** | | |
| --- | --- | --- | --- | --- | --- |
| **Chr** | **Test** | **Reference** | **Chr** | **Test** | **Reference** |
| **19** | 1,363 | 1,358 | **19** | 1,392 | 1,405 |
| **X** | 1,342 | 1,339 | **X** | 760 | 742 |
| **Y** | 128 | 133 | **Y** | 691 | 723 |

**Table S3.** Number CNAs found in memory CD4^+^ T cells from the popliteal lymph nodes of 10 NOD mice and 7 BALB/c mice infected with *L. major.*

| **Mouse Sample** | **Sex F/M** | **Age (w)^a^** | **Blood Glucose^b^** | **# of CNAs** |
| --- | --- | --- | --- | --- |
| Leish. NOD 1 | M | 9 | Low | 0 |
| Leish. NOD 2 | M | 9 | Low | 0 |
| Leish. NOD 3 | F | 14 | Low | 0 |
| Leish. NOD 4 | F | 14 | Low | 0 |
| Leish. NOD 5 | F | 14 | Low | 2 |
| Leish. NOD 6 | F | 14.2 | Low | 0 |
| Leish. NOD 7 | F | 14.2 | Low | 1 |
| Leish. NOD 8 | F | 14.2 | Low | 0 |
| Leish. NOD 9 | F | 14.2 | Low | 0 |
| Leish. NOD 10 | F | 14.2 | Low | 0 |
| Leish. BALB/c. 1 | F | 19 | Low | 0 |
| Leish. BALB/c. 2 | F | 19 | Low | 1 |
| Leish. BALB/c. 3 | F | 19 | Low | 0 |
| Leish. BALB/c. 4 | F | 19 | Low | 0 |
| Leish. BALB/c. 5 | F | 19 | Low | 1 |
| Leish. BALB/c. 6 | F | 19 | Low | 2 |
| Leish. BALB/c. 7 | F | 19 | Low | 0 |

### ^a^ Age is age of authorization after three weeks of infection

### ^b^ Glucose level measured in mmol/L

**Table S4.** Primer and Probes used for MLPA experiment. LPO: Left probe oligonucleotide; LHS: Left hybridizing sequence; RPO: Right hybridizing sequence, RPO: Right Probe oligonucleotide. Len: number of nucleotides

| **Gene** | **IFNG** | **CanX** | **Ascc3** | **Sod3** | **Sepsecs** | **Fancb (Chr X)** |
| --- | --- | --- | --- | --- | --- | --- |
| **LPO** | GGGTTCCCTAAGGGTTGGA | GGGTTCCCTAAGGGTTGGA | GGGTTCCCTAAGGGTTGGA | GGGTTCCCTAAGGGTTGGA | GGGTTCCCTAAGGGTTGGA | GGGTTCCCTAAGGGTTGGA |
| **LHS** | TCCAGCCTCAGGAAGCGGAAAAGGAGTCG | GTCCTAGGAGAGGGCCTGGTGTACCTTAG | TGACGGACTCTGTTGGTTACATGGAGACA | GATGCTGCCGAGATGCATGCAATCTGCAGGG | CTGTGCCTCTTGGGAACGTGCAAACTGTGAG | GGAGAACAGATAATAGGGCCATGACATTTTTAGGGAGAAGG |
| **RHS** | CTGCTGATTCGGGGTGGGGAAGAGATTGT | GTGGGAGCTCAAAACCTCAAGATGTCACC | AAGGCTATCAGACAGACGTTTGGTCCCTT | TACAACCATCAGCCACGCTGCCACCGGATCA | TGGCCATACTTTTCGAGGCTTCATGTCCCAT | GCCAAAATCCGTCAGTCCAAGCGCAAAGTTCAGAGAGAAAG |
| **RPO** | TCTAGATTGGATCTTGCTGGCAC | TCTAGATTGGATCTTGCTGGCAC | TCTAGATTGGATCTTGCTGGCAC | TCTAGATTGGATCTTGCTGGCAC | TCTAGATTGGATCTTGCTGGCAC | TCTAGATTGGATCTTGCTGGCAC |
| **LHS len** | 29 | 29 | 29 | 31 | 31 | 41 |
| **RHS len** | 29 | 29 | 29 | 31 | 31 | 41 |
| **Hs len** | 58 | 58 | 58 | 62 | 62 | 82 |
| **Total len** | 100 | 100 | 100 | 104 | 104 | 124 |
